# White Matter Hyperintensities in Older Adults and Motoric Cognitive Risk Syndrome

**DOI:** 10.17756/jnpn.2016-009

**Published:** 2016-11-03

**Authors:** Joanna L. Mergeche, Joe Verghese, Gilles Allali, Cuiling Wang, Olivier Beauchet, V.G. Pradeep Kumar, P.S. Mathuranath, Jennifer Yuan, Helena M. Blumen

**Affiliations:** 1 Department of Neurology, Albert Einstein College of Medicine, Bronx, NY 10461, USA; 2 Department of Medicine, Albert Einstein College of Medicine, Bronx, NY 10461, USA; 3 Department of Clinical Neurosciences, Geneva University Hospitals, Geneva, Switzerland; 4 Departments of Epidemiology, Albert Einstein College of Medicine, Bronx, NY 10461, USA; 5 Department of Neurosciences, Angers University Hospital, Angers, France; 6 Department of Neurology, Baby Memorial Hospital, Kozhikode, Kerala, India; 7 Department of Neurology, National Institute of Mental Health & Neurosciences, Bengaluru, Karnataka, India

**Keywords:** White matter hyperintensities, Motoric cognitive risk, Gait, Cognition, Cerebrovascular disease, Vascular injury

## Abstract

**Introduction:**

Motoric cognitive risk (MCR) syndrome is a recently described pre-dementia syndrome characterized by slow gait and cognitive complaints that has been implicated as a predictor of cognitive decline and dementia in older adults. Previous work suggests that cerebrovascular disease is associated with MCR. White matter hyperintensities (WMH) are postulated to be a product of cerebrovascular disease, and have been associated with impaired mobility and impaired cognition. This study aimed to determine if MCR is associated with regional WMH.

**Methods:**

Two cross-cultural cohorts of non-demented older adults were examined: 174 from a French memory clinic (62.1% male, mean age 70.7 ± 4.3 years) and 184 from an Indian community-dwelling cohort (55.4% male, mean age 66.2 ± 5.2 years). Participants were evaluated for slow gait, cognitive complaints, and regional WMH via MRI (fluid attenuated inversion recovery) FLAIR sequence.

**Results:**

Overall, 20.7% of participants met criteria for MCR, and 72.9% of participants had WMH on FLAIR. WMH in the frontal, parieto-occipital, temporal, basal ganglia, cerebellum, or brainstem were not associated with MCR in either of the two cohorts.

**Conclusion:**

WMH was not significantly associated with MCR in this studied sample of participants, suggesting that other cerebrovascular pathophysiological mechanisms, or combination of mechanisms, might underlie MCR.

## Introduction

Motoric cognitive risk (MCR) syndrome is a recently described pre-dementia syndrome characterized by slow gait and cognitive complaints [1–3]. MCR has been implicated as a predictor of cognitive decline and dementia in older adults and has been associated with increased mortality [2, 4]. MCR can be easily assessed in a variety of clinical settings without complex or extensive testing. As such, MCR allows for optimized assessment of dementia risk, initiation of early preventative measures, reduced healthcare costs, and potentially reduced mortality [4–6].

MCR has been shown to predict both Alzheimer’s disease [3] and vascular dementia [1] in previous cohort studies. Cerebrovascular disease is a clear contributor to vascular dementia, but has also been implicated in the pathogenesis of Alzheimer’s dementia [7]. MCR and cerebrovascular disease share many risk factors such as age, hypertension, and diabetes [3, 8–13]. A previous study evaluated regional lacunar infarcts and cortical microbleeds as measures of cerebral small vessel disease in the context of MCR [14] frontal lacunar infarcts were associated with MCR while cortical micro bleeds were not associated with MCR in this study. These findings suggest that specific types of regional cerebrovascular injury might contribute to the evolution of MCR.

White matter hyperintensities (WMH) are areas with high signal intensities on fluid attenuated inversion recovery (FLAIR) magnetic resonance images. WMH are ubiquitous in aging populations and have often been regarded as non-specific. The pathophysiological evolution of WMH is hypothesized to be a product of cerebrovascular disease and reduced vascular integrity [15, 16]. Risk factors for cerebrovascular disease, such as hypertension, diabetes, and aging, have been associated with the development of WMH [17, 18]. WMH are associated with many clinical consequences like impaired mobility and impaired cognition [19–22]. Rosario et al. found that microstructural integrity is a moderating factor in the association between WMH and gait [23]. Two studies identified associations between regional WMH and mild cognitive impairment syndrome, another pre-dementia syndrome [18, 24]. Interestingly, cerebral blood flow studies support that the pathogenesis and clinical presentation of WMH vary depending on the topographic location of the WMH in the brain [25].

No studies have assessed whether regional WMH, as a measure of cerebrovascular disease, are associated with MCR. Considering previous work suggesting that MCR is predictive of vascular dementia [1, 26], that cerebrovascular disease shares common risk factors with MCR and WMH [1, 3], and that regional WMH have cognitive consequences [3, 4, 20, 21], we hypothesized that MCR would be associated with regional WMH perhaps in the frontal region, as found previously for lacunar infarcts [14]. In this study, we evaluate whether MCR is associated with regional WMH in two large cohorts of non-demented older adults from a developed country, France, and lower-middle income country, India.

## Materials and Methods

### Participants

Participants were recruited from two independent studies in a developed country, France, and lower-middle income country, India. These two cohorts were chosen for this analysis as they had previously participated in MCR collaborative studies [3, 14, 27, 28], have neuroimaging available, and previous studies have indicated that there may be differences in prevalence of cerebrovascular disease by country and ethnicity [29–31]. The French cohort (N = 174) was obtained from a memory clinic population at Angers University Hospital; they were recruited in the Gait and Alzheimer Interactions Tracking study, an ongoing cross-sectional study in France. The Indian cohort (N = 184) is a community-dwelling cohort from the Kerala-Einstein study [14, 27, 28], and was recruited from Neurology clinics at Baby Memorial Hospital in Kozhikode in the southern Indian state of Kerala. All 358 participants were >60 years of age, were non-demented [27], and had complete quantitative gait measurements, cognitive complaint data, neurologic examination, and FLAIR imaging. Exclusion criteria were history of dementia, presence of severe audiovisual disturbances, severe medical or neurological disease, and standard MRI exclusion criteria, as previously described [27].

Written informed consent was obtained from all participants. The parent studies received approval from their local institutional review boards. The Albert Einstein College of Medicine Institutional Review Board (Bronx, NY, USA) approved this analysis.

### Motoric cognitive risk syndrome (MCR)

MCR is defined by slow gait and cognitive complaints in the absence of dementia and mobility disability, as previously described [1, 3]. Cognitive complaints were elicited from participants in clinician interviews and from standardized questionnaires [32–34]. Slow gait was defined as walking speed one standard deviation below the age- and sex-adjusted means during normal pace walking condition. These adjusted means were previously established in each cohort [3]. The slow gait threshold scores in the Indian cohort were: males <75 years at 79.08 cm/s, males >75 years at 54.55 cm/s, females <75 years at 72.00 cm/s, and females >75 years at 44.45 cm/s. The slow gait threshold scores in the French cohort were: males <75 years at 94.09 cm/s, males >75 years at 84.26 cm/s, females <75 years at 92.02 cm/s, and females >75 years at 74.66 cm/s. Gait speed was measured quantitatively with a computerized walkway with embedded pressure sensors (GAITRite, CIR systems, Havertown, PA, USA) for all participants in the French cohort and for 149 participants of the Indian cohort [35]. In the Indian cohort, 35 participants had gait speed measured over a fixed distance, and then converted to centimeters per second [1, 3, 36]. Dementia was diagnosed at consensus case conferences in both cohorts using established criteria. See mobility disability criteria in previous papers [2]. If participants met criteria for both slow gait and cognitive complaint, and did not have dementia or mobility disability, they were designated as having MCR.

### Fluid attenuated inversion recovery (FLAIR)

Participants underwent a standard cerebral MRI protocol including a FLAIR sequence. FLAIR sequences are particularly sensitive to WMH as they mask the cerebrospinal fluid that can otherwise cloud other MRI images and findings. Images were acquired in France (University of Angers Hospital in Angers) and India (Baby Memorial Hospital in Kerala) and sent to Albert Einstein College of Medicine (Bronx, NY, USA) for image rating and analysis. Imaging was performed at both sites with a 1.5-Tesla MRI scanner (Magnetom Avanto, Siemens Medical Solutions, Erlangen, Germany) using a standard MRI protocol including FLAIR images (acquisition matrix = 256 × 232, FOV = 240 mm × 180 mm, resolution = 5 mm, gap = 0.5 mm, 30 slices, TE/TR/TI = 122 ms/9000 ms/2500 ms).

### White matter hyperintensities ratings

FLAIR images were rated using the well-established rating scale for WMH derived by Wahlund and colleagues [37]. Ratings were made for six brain regions: frontal, parieto-occipital, temporal, basal ganglia, cerebellum, and brainstem. Bilateral ratings were obtained for all regions except for the brainstem, which was rated as one region given its small size and difficulty of establishing laterality for medial lesions. In the frontal, parieto-occipital, temporal, cerebellum, and brainstem regions, focal lesions >2 mm were rated a 1, confluent lesions were rated a 2, and diffuse involvement of the entire region was rated a 3. In the basal ganglia, focal lesions ≥5 mm were rated a 1, >1 focal lesion was rated a 2, and confluent lesions were rated a 3. Regional WMH ratings were analyzed as binomial data: present (inclusive of focal, confluent, and diffuse WMH) or absent. Two raters, blinded to clinical information and MCR status performed all WMH ratings (J.L.M, N = 322 and J.Y., N = 26). Inter-rater reliability was calculated between the raters and a board-certified neurologist (G.A.) and a board-certified radiologist (C.K.) for a subset of images (J.L.M., κ = 0.78 for 30 images and J.Y., κ = 0.67 for 16 images, indicating adequate agreement according to conventional guidelines of inter-rater agreement [38]). Strokes detected on FLAIR were confirmed with the board-certified neurologist.

### Covariates

Participant characteristics were obtained from the independent studies and included age, sex, years of education, history of stroke and/or stroke on MRI, diabetes, and hypertension. Smoking history was only collected in the Indian cohort. Body mass index (BMI) was only collected in the French cohort.

### Statistical analyses

Statistical analyses were performed using JMP 10 software (SAS Institute Inc., Cary, NC, USA) and STAT12 (StataCorp LP, College Station, TX, USA). Cohort characteristics between subjects with and without MCR were compared using Student t-test test for continuous variables, and Fisher exact test or Pearson χ^2^ tests for categorical variables where appropriate. P-values ≤.05 were considered significant.

## Results

### Participant characteristics

Table 1 summarizes participant characteristics in the French and Indian cohorts. The French cohort was significantly older (70.7 ± 4.3 years vs. 66.2 ± 5.2 years, P<0.001), had more education >13 years (24.1% vs. 6.2%, P<0.001), less diabetes (1.7% vs. 32.6%, P<0.001), less hypertension (30.5% vs. 63.0%, P<0.001), and faster gait velocity (109.4 ± 20.3 cm/s vs. 88.8 ± 22.8 cm/s, P<0.001) than the Indian cohort.

### MCR

The prevalence of MCR in this study was 20.7%. The prevalence of MCR was similar between the French and Indian cohorts: 19.5% in the French cohort and 21.7% in the Indian cohort (P = 0.61). In the French cohort, participants with and without MCR were comparable in patient characteristics as shown in Table 1. In the Indian cohort, participants were comparable in all characteristics except that patients with MCR were more likely to be male (75.0% vs. 50.0%, P = 0.004). In the French cohort, patients with MCR had significantly higher BMI (28.1 ± 5.0 vs. 25.6 ± 3.2, p<0.001).

### WMH

The prevalence of WMH overall in this study was 72.9%. Participants with and without WMH were comparable in terms of sex, education, stroke, diabetes, and hypertension. The French cohort had higher prevalence of WMH overall than the Indian cohort (77.5% vs. 68.5%, P = 0.05). In the pooled sample, participants with WMH were older than participants without WMH (69.2 ± 5.5 years vs. 66.2 ± 4.0 years, P<0.001).

When evaluating WMH in the context of the components of MCR, there were no significant associations. In the French cohort, 17.0% of patients with slow gait had WMH vs. 28.2% of patients without slow gait, p = 0.13. In the Indian cohort, 29.4% of patients with slow gait had WMH vs. 24.2% of patients without slow gait, p = 0.46 and 65.9% of patients with cognitive complaint had WMH vs. 58.6% without cognitive complaint, p = 0.35.

**Figure 1:**
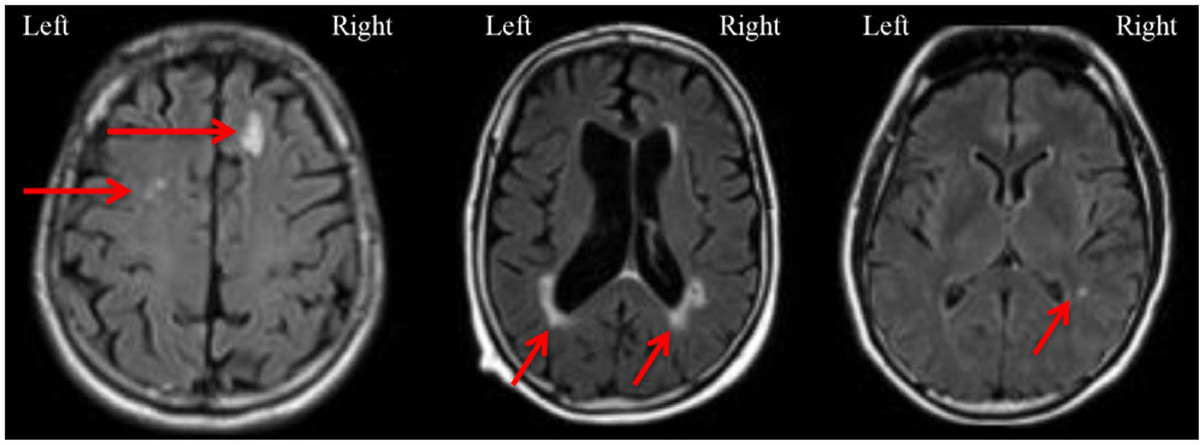
WMH on FLAIR. This figure presents FLAIR image examples of WMH in the frontal region (top), the parieto-occipital region (middle), and temporal region (bottom). Right and left are indicated for orientation. Red arrows indicating WMH on the images.

The location of the most prevalent regional WMH was in the right frontal (French 67.2% and Indian 60.0%) and left frontal (French 61.5% and Indian 56.0%) regions, as demonstrated in Table 2. The least prevalent regional WMH were in the right cerebellum (French 2.3% and Indian 0%) and left cerebellum (French 0.6% and Indian 2.7%). There were no significant differences in the prevalence of regional WMH between patients with and without MCR as shown in Table 2. There were no associations between the severity of WMH overall or in specific regions with MCR (data not shown). Figure 1 depicts FLAIR images of WMH in the frontal, parieto-occipital, and temporal regions.

## Discussion

This study is the first to evaluate regional WMH in the context of the MCR syndrome in effort to clarify the contribution of cerebrovascular disease and vascular injury to the pathophysiology of MCR. The prevalence of MCR in this study was within the range of previously published MCR studies [1, 2, 4, 14], but higher than the estimated global prevalence [3]. This study also found that BMI was significantly associated with MCR in the French cohort, as demonstrated in previous work [3]. The prevalence of WMH was also consistent with previous studies [14, 22, 24, 39–44]. However, the French cohort did have a higher prevalence of WMH overall. The higher prevalence of WMH in the French cohort might be due to the fact that the cohort was obtained from a memory clinic, which by definition means all of these patients had cognitive complaint. The Indian cohort demonstrates that a greater proportion of patients with cognitive complaint had WMH than patients with slow gait. These findings suggest that perhaps WMH contribute to the individual components of the MCR syndrome, as previously suggested by other studies [19–22], but additional pathology or mechanisms might be required to cross the diagnostic threshold that presents as the MCR syndrome.

The primary finding of this study is that the association of regional WMH with MCR is not statistically significant in this studied sample and that WMH are rather a ubiquitous finding in non-demented older adults. This finding suggests that vascular mechanisms other than WMH may contribute to the pathophysiology of MCR, as was previously suggested in the case of regional lacunar infarcts [14]. Wang et al. [14] reported that WMH overall were not associated with MCR and our study replicated these findings. Our study further evaluated this by assessing regional WMH in the context of MCR in two independent cohorts of non-demented older adults.

Cerebrovascular disease and vascular injury are products of different pathophysiological mechanisms. For example, microbleeds are small foci of chronic blood products in otherwise normal brain tissue [45]. Anatomic and physiolopathologic studies suggest that most WMH have a chronically ischemic pathogenesis and are a product of widespread cerebrovascular disease and reduced vascular integrity [15, 16]. Lacunar infarcts, however, are the product of a subcortical ischemic lesion at the level of a deep perforating artery secondary to an occlusion [46, 47]. Perhaps the ubiquitous presence of WMH present in the vast majority of older non-demented adults are a general marker for cerebrovascular disease, and the relatively less common lacunar infarct is a more specific marker for MCR when present in specific regions like the frontal lobe [14]. WMH are a less specific form of cerebrovascular injury than lacunar infarcts. WMH might also be due to neurodegenerative or age-related changes, which might also explain the lack of significance in their relation to MCR.

Some limitations should be considered. The cross-sectional nature of these data left us unable to establish causality. The design also did not allow us to confirm which participants develop dementia and if so, what subtype of dementia as the cohorts were part of parent studies that do not include longitudinal follow-up assessments. A longitudinal follow up should be included in future studies to track development of dementia and progression of WMH. Smoking history and BMI were not collected in both cohorts. WMH were not measured with an automatic measure that could provide a more objective measure and metric information such as volume; this study’s evaluation of WMH was based solely on manual quantification. Lastly, the characterization of MCR is a clinical concept that lacks definitive pathophysiological correlates and biomarkers; it is certainly a consideration that neurodegenerative conditions such as Parkinson’s disease or other Parkinsonian syndromes that include motor and cognitive components could contribute to some of the findings in our patient population. For example, a patient in the early stages of Parkinson’s disease could present with similar features prior to being clinically diagnosed with Parkinson’s disease and also have WMH [48, 49]. In conclusion, the findings of this study suggest that small vessel disease with risk factors related to MCR other than WMH should be examined. The pathophysiology of MCR remains unclear, but this study is important in that it contributes data to one of the hypotheses. It is reasonable that other vascular risk factors, or combination of risk factors, be investigated further to better understand the mechanism behind the development MCR and find points of potential prevention.

## Figures and Tables

**Table 1:** Participant Characteristics^1^.

	All	No MCR	MCR	P-value^2^

**French Cohort**				

N	174	140	34	

Age M ± SD, years	70.7 ± 4.3	70.6 ± 4.4	71.0 ± 4.0	0.62

Sex, male	62.1%	60.7%	67.7%	0.45

Body Mass Index	26.1 ± 3.8	25.6 ± 3.2	28.1 ± 5.0	<0.001

Education				0.28
≤4 years	1.7%	2.1%	0%	
5–8 years	39.1%	37.1%	47.1%	
9–12 years	35.1%	34.3%	38.2%	
≥13 years	24.1%	26.4%	14.7%	

Stroke	4.6%	5.0%	2.9%	0.59

Diabetes	1.7%	2.1%	0%	0.25

Hypertension	30.5%	29.3%	35.3%	0.50

Gait Velocity M ± SD, cm/s	109.4 ± 20.3	116.1 ± 15.6	81.5 ± 12.0	<0.001

**Indian Cohort**				

N	184	144	40	

Age M ± SD, years	66.2 ± 5.2	65.9 ± 5.2	67.4 ± 4.9	0.09

Sex, male	55.4%	50.0%	75.0%	0.004

Education				0.10
≤4 years	5.6%	7.2%	0%	
5–8 years	35.4%	25.8%	9.6%	
9–12 years	52.8%	42.1%	10.7%	
≥13 years	6.2%	4.5%	1.7%	

Stroke^2^	7.1%	5.6%	12.5%	0.16

Diabetes	32.6%	30.6%	40.0%	0.27

Hypertension	63.0%	61.1%	70.0%	0.30

History of Smoking	19.0%	18.8%	20.0%	0.86

Gait Velocity M ± SD, cm/s	88.8 ± 22.8	96.2 ± 19.4	62.0 ± 11.0	<0.001

1 Data presented as percentages or mean ± standard deviations.

2 Student t-test for continuous variables, and Fisher exact test or Pearson χ^2^ tests for categorical variables.

**Table 2:** Regional WMH and MCR^1^.

	All	No MCR	MCR	P-value^2^

**French Cohort**				

N	174	140	34	

White Matter Hyperintensities Present^4^	77.6%	80.0%	67.7%	0.13

Right Frontal	67.2%	69.3%	58.8%	0.25
Left Frontal	61.5%	62.9%	55.9%	0.46
Right Parieto-Occipital	44.3%	44.3%	44.1%	0.99
Left Parieto-Occipital	47.1%	48.6%	41.2%	0.44
Right Temporal	7.5%	6.4%	11.8%	0.31
Left Temporal	5.8%	5.0%	8.8%	0.41
Right Basal Ganglia	6.9%	6.4%	8.8%	0.63
Left Basal Ganglia	7.5%	8.6%	2.9%	0.22
Right Cerebellum	2.3%	2.9%	0%	0.18
Left Cerebellum	0.6%	0.7%	0%	0.51
Brainstem	5.2%	3.6%	11.8%	0.08

**Indian Cohort**				

N	184	144	40	

White Matter Hyperintensities Present^4^	68.5%	66.7%	75.0%	0.31

Right Frontal	59.8%	57.6%	67.5%	0.26
Left Frontal	56.0%	55.6%	57.5%	0.83
Right Parieto-Occipital	45.1%	45.1%	45.0%	0.99
Left Parieto-Occipital	44.0%	45.1%	40.0%	0.56
Right Temporal	6.5%	6.9%	5.0%	0.65
Left Temporal	7.1%	8.3%	2.5%	0.16
Right Basal Ganglia	6.0%	5.6%	7.5%	0.66
Left Basal Ganglia	5.4%	4.9%	7.5%	0.53
Right Cerebellum	0%	0%	0%	-
Left Cerebellum	2.7%	3.5%	0%	0.12
Brainstem	3.3%	3.5%	2.5%	0.75

1 Data presented as percentages.

2 Pearson χ^2^ tests for categorical variables.

## Abbreviations

FLAIR: Fluid attenuated inversion recovery
MCR: Motoric cognitive risk
WMH: White matter hyperintensities
